# Availability and affordability of anti-VEGF biosimilars for the treatment of age-related macular degeneration and diabetic macular oedema in Sri Lanka

**Published:** 2025-01-31

**Authors:** Mapa Prabhath Piyasena, Mangala Dhanapala

**Affiliations:** 1 Deputy Director: Vision and Eye Research Institute, Faculty of Health, Medicine and Social Care, Anglia Ruskin University, UK.; 2 Consultant Vitreo-Retinal Surgeon: Retina Research Unit, National Eye Hospital, Colombo, Sri Lanka.

**In Sri Lanka, anti-VEGF biosimilars are available free of cost in the public eye health care system for treatment of macular diseases.**

With the rapidly ageing population and increasing prevalence of diabetes mellitus (the latest population-based survey reports the age-standardised prevalence of diabetes as 21.8%^1^), the most common macular diseases among adults in Sri Lanka are diabetic macular oedema and age-related macular degeneration.

The eye care in Sri Lanka is provided by the public sector, private sector, and non-governmental organisations (‘third sector’). The public sector provides all aspects of eye care services, including anti-vascular endothelial growth factor (anti-VEGF) injections, free of charge to the citizens of Sri Lanka. The medical supplies division of the ministry of health in Sri Lanka provides the required number of anti-VEGF injections island-wide to those tertiary and secondary level health care institutions where a specialist general ophthalmologist or a specialist vitreo-retinal surgeon is available. In Sri Lanka, many general ophthalmologists perform retinal procedures; these ophthalmologists can therefore administer anti-VEGF injections to patients. Intravitreal injections are administered in the main operating theatres under strict sterile conditions.

**Figure 1:**
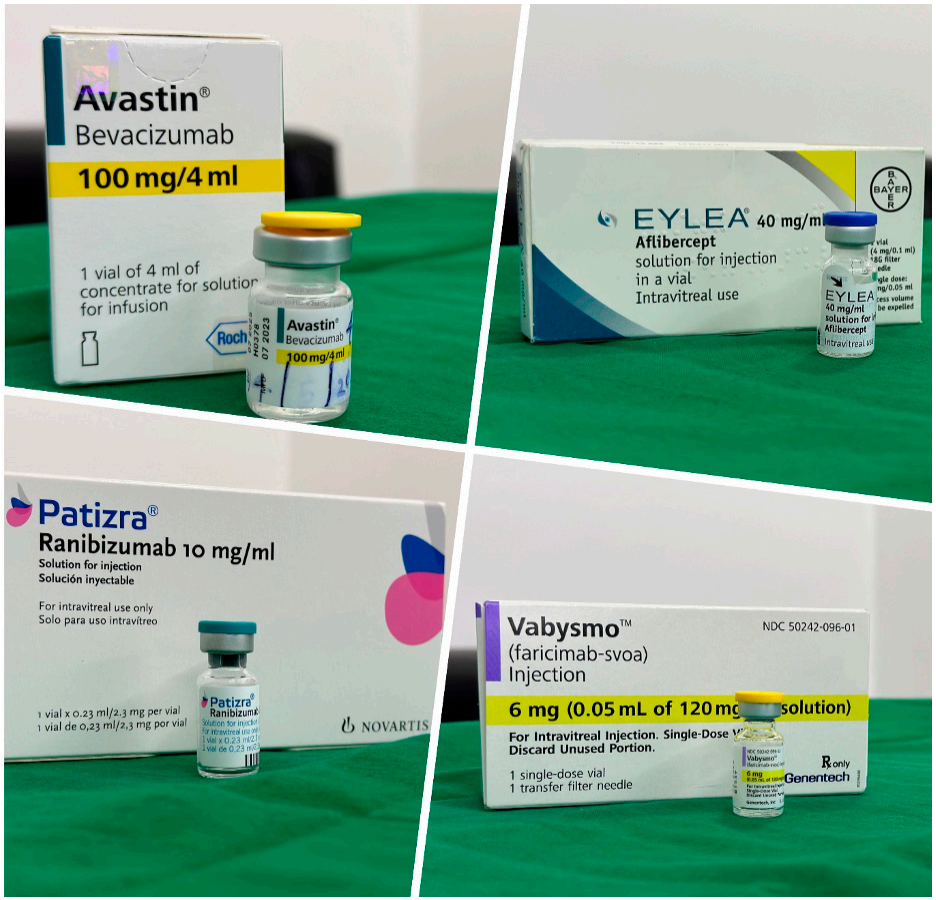
Vials of anti-VEGF injections available in Sri Lanka.

Intravitreal anti-VEGF injections are an effective main mode of treatment for macular diseases globally.^2,3^ Most of the anti-VEGF biosimilars used worldwide are also available in Sri Lanka. The commonly used ones are bevacizumab (Avastin), ranibizumab (Lucentis), aflibercept (Eyelea), and faricimab (Vabysmo). Biosimilar bevacizumab preparations available in Sri Lanka are Avegra (by Biocad) and Abevmy (by Biocon Ltd). The biosimilar aflibercept that is available is Zaltrap (by Regeneron). Original ranibizumab is available, in addition to Patizra, which is the ranibizumab preparation made available for low- and-middle-income countries by Novartis Indonesia (Figure 1).

© DR MANGALA DHANAPALA, SRI LANKA CC BY-NC-SA 4.0

Public sector hospitals in Colombo, which cover the Greater Colombo area, carry out around 2,000 anti-VEGF injections per month, while the private sector hospitals catering for this area also provide roughly the same number (approximately 24,000 injection doses per year in a district with 500,000 people with diabetes). Out of these, around 90% of the injections are Avastin, with the remaining 10% comprising Patizra, Eyelea, and Vabysmo. In clinical practice one Avastin vial is shared among 30 to 40 patients, hence the cost of an injection in the public sector is around LKR 4,000 (roughly US $13.50) per patient. It is given free of charge to the patient. In the private sector, the cost per Avastin injection ranges from LKR 16,000 to LKR 35,000 (roughly US $53 to $117); per Patizra injection, from LKR 90,000 to LKR 125,000 (roughly US $300 to $417); and per Vabysmo injection, from LKR 300,000 to LKR 325,000 (roughly US $1,000 to $1,084). The problems with regard to the use of biosimilar anti-VEGF agents in Sri Lanka pertain to the cost of the products (the prices are very close to that of the original product) and the lack of designated local suppliers for them. These are the probable reasons why these products are not very popular amongst retina specialists.

**Figure 2:**
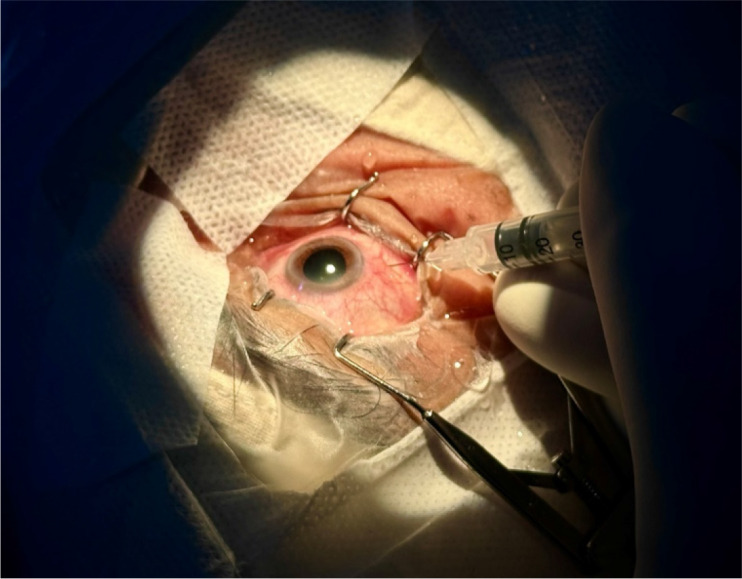
An image of treating a patient with an Intra-vitreal injection in an operating theatre in Sri Lanka.

© DR MANGALA DHANAPALA, SRI LANKA CC BY-NC-SA 4.0

Patients can choose the private sector for anti-VEGF care, in which case the hospital charges and cost of medicines or procedures have to be paid out of pocket. If a patient cannot afford anti-VEGF care in the private sector, they will be referred to the nearest secondary level or tertiary level public sector specialist eye unit, without the need for a referral letter from a general practitioner, as is the case in some high-income countries. This has improved access to anti-VEGF injections.

**Two common approaches after the monthly loading dose**

**1. Treat and extend.** If the macula stays dry, the time between injections is gradually increased by two weeks, up to 12 weeks. However, every visit includes an injection to prevent swelling from returning.

**2. Pro re nata (PRN) or ‘as needed’.** Patients are checked monthly, and injections are given only if swelling (oedema) reappears.

For some patients, if the swelling doesn't improve after the initial three doses, doctors may recommend three more monthly injections.

These methods help tailor the treatment to the patient's needs while balancing effectiveness and the number of visits required. However, many patients, especially those on low incomes, find it hard to follow these schedules due to the financial burden of missing work for frequent visits.

The issue of lack of adherence to prescribed anti-VEGF treatment in Sri Lanka has not been scientifically studied. Anecdotal evidence shows that most of the barriers are associated with a lack of awareness of the importance of treatment, difficulties in managing travel logistics, not having someone to accompany the patient to appointments, or fear and anxiety about injections.

The recommended treatment of a monthly spaced loading dose, followed by a treat and extend or PRN (pro re nata) regimen, is not strictly followed by the patients, especially those from low socio-economic backgrounds, because losing a day's wages (to receive treatment) is a significant burden on patients and their families.
